# Draft Genome Sequence of Leptospira yasudae Strain BJ3, Isolated from the Soil of an *Ex Situ* Wild Animal Conservation Area

**DOI:** 10.1128/MRA.00723-21

**Published:** 2021-09-30

**Authors:** Nadia Aqilla Shamsusah, Hani Kartini Agustar, Fairuz Amran, Rozita Hod

**Affiliations:** a Department of Earth Sciences and Environmental, Universiti Kebangsaan Malaysia, Bangi, Malaysia; b Bacteriology Unit, Institute for Medical Research, Kuala Lumpur, Malaysia; c Department of Community Health, Universiti Kebangsaan Malaysia, Cheras, Malaysia; Indiana University, Bloomington

## Abstract

Previously, a novel *Leptospira* strain (BJ3) was isolated from the soil of an *ex situ* wild animal conservation area in Perak, Malaysia. Molecular identification via whole-genome sequencing confirmed that the strain was Leptospira yasudae. Here, we report the draft genome sequence of *L. yasudae* strain BJ3.

## ANNOUNCEMENT

Leptospirosis is a zoonotic disease caused by helical bacteria from the genus *Leptospira* (1). This disease has spread throughout the world, especially in the tropical and subtropical regions (2). Leptospirosis can be transmitted either through direct contact with the urine of infected animals or indirectly through contaminated soil/water (3). Previously, a strain of *Leptospira* (BJ3) was isolated from the soil of an *ex situ* wild animal conservation area in Perak, Malaysia (4). Based on the classification of its 16S rRNA gene, the isolate was deemed a novel species named Leptospira yasudae and was classified under the pathogenic group. Infection in wildlife might result in the introduction of new serovars to humans and endangered species, hence hampering the success of wildlife conservation. In the present study, we conducted whole-genome sequencing to further characterize the strain. Here, we present the draft genome sequence of *L. yasudae* strain BJ3.

The bacteria were first cultured and maintained in liquid Ellinghausen-McCullough-Johnson-Harris (EMJH) medium at 30°C and observed weekly under a darkfield microscope. To obtain a pure single isolate, one small drop of the EMJH culture was spread onto an EMJH agar plate. After 7 days, a single colony was picked and cultured in fresh liquid EMJH medium. Once the culture reached its maximum density of ∼1 × 10^8^ leptospires/ml, genomic DNA (gDNA) was extracted using a Presto mini gDNA bacteria kit (Geneaid Biotech, Taiwan), based on the manufacturer’s protocol. Sequencing was performed at 1st BASE Laboratories, Malaysia. Sequencing libraries were generated using a NEBNext Ultra DNA library prep kit for Illumina (NEB, USA) and sequenced on a HiSeq 4000 system (Illumina, USA) to obtain 2 × 150-bp reads. Sequence adaptors and reads with low-quality scores were removed using BBDuk in the BBTools package (5). The read quality was then assessed using FastQC v0.11.3 (6). Quality control (QC) reads were assembled *de novo* using SPAdes v3.11.1 (7). The resulting >1,000-bp contigs (contiguous sequences) then underwent a search using BLAST against the National Center for Biotechnology Information (NCBI) nucleotide database (https://blast.ncbi.nlm.nih.gov/). For certain scaffolds with low coverage (<100× coverage) and the best BLAST result for nontarget (non-*Leptospira*) organisms, the contigs were removed because they were likely sequence contaminants. The genome statistics were assessed using QUAST v4.6.0 (8); finally, the contigs were annotated using the NCBI Prokaryotic Genome Annotation Pipeline (PGAP) as per the GenBank submission (9). The tRNAs and rRNAs were predicted using tRNAscan-SE v1.3.1 (10) and RNAmmer v1.2 (11). Default parameters were used for all software. To characterize the serogroup/serovar of the isolate, multilocus sequence typing (MLST) analysis scheme 1 (12) was performed and compared with the PubMLST database (https://pubmlst.org/). Moreover, traditional serotyping (microscopic agglutination test) was performed at the Institute for Medical Research (IMR), Malaysia, and the Pasteur Institute, France, against 46 local and global antiserum samples.

The data set comprised 6,343,849 paired-end reads and a raw coverage depth of 213×. *De novo* assembly resulted in 4,537,672 bases with 26 contigs and an *N*_50_ value of 642,216 bp. The *L*. *yasudae* strain BJ3 draft genome comprises ∼4.54 Mbp, with an overall GC content of 45.23%. In total, there were 4,134 coding sequences (CDS), 2 5S rRNAs, 1 16S rRNA, 1 23S rRNA, and 59 pseudogenes. Partial sequencing of the 16S rRNA gene indicated that *L. yasudae* strain BJ3 is closely related to *L. kmetyi* strain Bejo-Iso9. For MLST analysis, *L. yasudae* strain BJ3 was assigned a new sequence type (ST) number (ST262), as no match was found for its serogroup/serovar in the PubMLST database, suggesting that it might contain a new kind of serogroup/serovar. Traditional serotyping also showed negative reactions against the 46 local and global antiserum samples tested, thus supporting our MLST findings. A detailed report of the genomic data analysis, in the context of virulence and unique characteristics, will be provided in future publications. The availability of the *L. yasudae* strain BJ3 genome sequence could yield much information, thus enhancing our understanding of its role as a new local pathogen of humans and wildlife.

### Data availability.

This whole-genome project has been deposited in the NCBI GenBank database under accession number JACCKC000000000. The BioProject accession number is PRJNA624226, and the BioSample accession number is SAMN14572009. The Illumina HiSeq raw data have been deposited in the NCBI Sequence Read Archive (SRA) under accession number SRP332200.
